# Modeling predictors of risky drug use behavior among male street laborers in urban Vietnam

**DOI:** 10.1186/1471-2458-13-453

**Published:** 2013-05-07

**Authors:** Van Huy Nguyen, Michael P Dunne, Joseph Debattista

**Affiliations:** 1 Department of Health Management and Organization, Institute for Preventive Medicine and Public Health, Hanoi Medical University, 01 Ton That Tung Str., Dong Da Dist., Hanoi, Vietnam; 2 School of Public Health and Social Work, Faculty of Health, Queensland University of Technology, Brisbane, Australia; 3 Brisbane Sexual Health and HIV Service, MetroNorth Hospital and Health Service, Brisbane, Australia

**Keywords:** Vietnam, Drug use, Risk Behavior(s), HIV/AIDS, Unskilled Laborer(s), IMB Model, Structural Equation Modeling (SEM)

## Abstract

**Background:**

The application of theoretical frameworks for modeling predictors of drug risk among male street laborers remains limited. The objective of this study was to test a modified version of the IMB (Information-Motivation-Behavioral Skills Model), which includes psychosocial stress, and compare this modified version with the original IMB model in terms of goodness-of-fit to predict risky drug use behavior among this population.

**Methods:**

In a cross-sectional study, social mapping technique was conducted to recruit 450 male street laborers from 135 street venues across 13 districts of Hanoi city, Vietnam, for face-to-face interviews. Structural equation modeling (SEM) was used to analyze data from interviews.

**Results:**

Overall measures of fit via SEM indicated that the original IMB model provided a better fit to the data than the modified version. Although the former model was able to predict a lesser variance than the latter (55% vs. 62%), it was of better fit. The findings suggest that men who are better informed and motivated for HIV prevention are more likely to report higher behavioral skills, which, in turn, are less likely to be engaged in risky drug use behavior.

**Conclusions:**

This was the first application of the modified IMB model for drug use in men who were unskilled, unregistered laborers in urban settings. An AIDS prevention program for these men should not only distribute information and enhance motivations for HIV prevention, but consider interventions that could improve self-efficacy for preventing HIV infection. Future public health research and action may also consider broader factors such as structural social capital and social policy to alter the conditions that drive risky drug use among these men.

## Background

Vietnam is one of a few countries in Asia and the Pacific region that is experiencing an exponential increase of HIV/AIDS among at-risk, drug-using populations [1]. The first case of HIV was reported in 1990 in Hochiminh City, but then rapidly increased among injection drug users (IDU). By 1999, 63 provinces reported more than 16,149 HIV-positive cases, of which 65% were IDU [1]. The same was also true in the most recent national data reporting that there have been 160,019 reported HIV cases and 44,050 deaths due to AIDS-related illnesses by the end of 2009, most (82.5%) were males with an overwhelming majority as IDU [2].

Although the HIV epidemic is primarily associated with injection drug use, its extent is highly variable across the country. In the cities of Hochiminh, Can Tho, Hai Phong, Thai Nguyen, and Quang Ninh, for instance, the HIV rate among IDU was over 40% [3]. In Hanoi, the first HIV infection was reported in 1993, but then increased rapidly among IDU from 3.3% in 1998 to 13.3% in 1999, 17.5% in 2000 [1], and 20.8% in 2008 [4].

Compared with nonmigrant populations, migrants are more vulnerable to risk behaviors for HIV. The separation from family, social disruption, breakdown of social networks, lack of social control and support and anonymity of urban living created opportunities for risk behaviors – substance abuse and risky sexual behaviors - placing them at particular risk for HIV infection [5]. A literature review by Voyer et al. [6] suggests that the variables of ethnicity, gender, marital status, mental health status, health perception, social support and access to health services were associated with drug use in most studies. According to Yang and Luo [5,7], in addition to migrants’ individual characteristics, such as education, marital status, and psychosocial well-being, that seem to have predisposed them to drug misuse, exposure to the social influence of drug-using peers, friends, or relatives in their social network may also facilitate migrants to take drugs. Whether drug use is examined separately or jointly with other risk behaviors, psychosocial well-being and behavior-specific social influences as measured in many studies are all significant risk factors, and their impacts are frequently consistent with the literature. Until now, although sexual risk behaviors among migrants have received greater attention, little is known about drug use behavior and its associated factors among this population [8].

**Figure 1:**
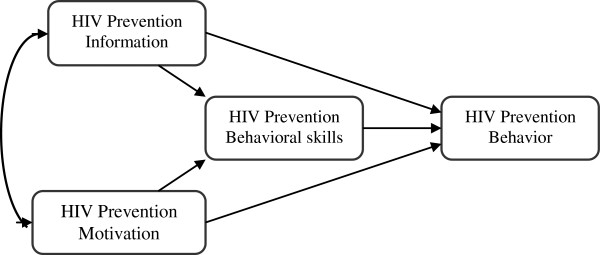
IMB model of HIV prevention behavior.

To identify an appropriate theory for the current study, a critical review of the literature is essential. As Edberg [9] argues, no theory is without its critique. Among the theories, the Information-Motivation-Behavioral Skills model (IMB) has been helpful and relevant to studies on HIV-related topics. Information is comprised of two sub-constructs (heuristic and transmission knowledge), motivation has three sub-constructs (attitudes, social norms and intentions), and behavioral skills has two or three sub-constructs depending upon research topics and populations. The model (see Figure 1) proposes that HIV preventive behavior of any kind is a function of HIV prevention information, HIV prevention motivation, and HIV prevention behavioral skills [10,11]. Specifically, HIV prevention information and motivation work through prevention behavioral skills to influence risk reduction behaviors, while both are also posited to have a direct impact on behavior [10]. In terms of its strengths, the model has been applied in prior studies to examine predictors of HIV risk behaviors among different populations within the context of both developed and developing countries [11-14]. Beyond its established strength in predicting, understanding, and informing interventions to change HIV risk behavior, the IMB model is viewed as a generalized approach to understanding and promoting health behavior [15]. However, this model also has some limitations. The focus on the psychological or individual-level factors limited the predictive power of behaviors. It has been argued that the model has been inconsistent in several populations, and may need further examination [16-18]. Given it has not reflected broader social factors, Odutolu [16] highlighted a need for its validation and adaptation in other populations and/or in other settings. Another approach is to conduct preliminary qualitative research in order to adapt or modify constructs within the standard model based on Aronowitz and Munzert’s recommendations [19]. Based on results of qualitative research, type of intervention and population, Aronowits and Muzert suggested adding some variables to constructs (information, motivaton and behavior skills) of the model [19].

In response to several of these recommendations, a recent qualitative study was conducted on this population which aimed to explore lived experiences of male migrants who served in informal sectors – performing unskilled, unregistered, and low-income labors within an urban setting of Vietnam [20]. The results highlighted some important social factors that placed these men at risk of HIV transmission. Family and community pressure, expectations and limited employment options in rural areas frustrated and compelled them to migrate great distances to the city for informal work. However, working in urban settings generated numerous stressors for these men, compelling them to seek out a range of coping strategies, such as sex and drug uptake. Risk behaviors for acquiring HIV, including unsafe sex and injection drug use, were more likely in men who had misperceptions of HIV/AIDS and experienced psychological stresses such as tedium, boredom, depression, fatalism, revenge, and family and social pressure as well as alcohol consumption than in other men. However, a key gap in the literature is a lack of quantitative research that can be statistically tested in order to validate previous qualitative findings and to identify the extent to what various factors have been considered to influence drug use behavior among this population.

The purpose of this study was to test a modified version of the IMB, which includes psychosocial stress, and to compare this modified version with the original IMB model for predicting risky drug use behavior among male street laborers, most of whom are migrant, minimally educated and unemployed. It was hypothesized that male street laborers who have better HIV prevention information and motivation and less psychosocial stress are less likely to be engaged in drug risk behavior. Psychosocial stress is a combination of four factors, mobility index, social isolation, depression and alcohol use (See Table 1 for details). In this study, we both adapted the existing constructs of the IMB model and added one more construct “psychosocial stress” to the model for testing its goodness-of-fit.

**Table 1:** The constructs of the original and modified IMB model

**Constructs**	**References**	**Number of items**	**Scale**	**Crobach’s α**
**The original model**				
***HIV preventive information***	[21,22]	**7**	True/false	0.63
*Transmission information*		3	True/false	0.66
*Heuristic information*		4	True/false	0.61
***HIV preventive motivation***	[23-25]	**21**	5-point semantic*¶*	0.91
*Attitudes*		7	5-point semantic*¶*	0.75
*Social norms*		7	5-point semantic§	0.83
*Intentions*		7	5-point semantic*†*	0.81
***HIV preventive behavioral skills***	[23,26]	**5**	5-point semantic#	0.86
*Skill 1 (preparation)*		3	5-point semantic#	0.76
*Skill 2 (practice)*		2	5-point semantic#	0.91
**The modified model**				
***3 constructs of the original model***				
***Psychosocial stress‡***		**4**	Different scales	0.71
*Mobility index‡*	[27]	2	-	-
*Social isolation*Ÿ	[28]	6	5-point	0.74
*Alcohol use¥*	[29]	2	-	0.60
*Depressionƒ*	[30-34]	10	4-point	0.88

Note: ¶ scale from 1 (negative evaluation) to 5 (positive evaluation); §scale from 1 (negative evaluation) to 5 (positive evaluation); †scale from 1 (very unlikely) to 5 (very likely); #scale from 1 (very hard) to 5 (very easy); ‡ the ratio of the number of migratory cities to years of total migration. (−) Not applicable as it is a ratio; Ÿscale from 0 (not at all) to 4 (almost always) during the past 4 weeks; ¥ a composite of the number of standard drinks and frequency of use over the past 4 weeks; ƒscale from 0 (rarely or none of the time) to 3 (most or all of the time) during the past week.

‡because the four indicators were associated with stresses given our qualitative findings [20,35] and statistic parameters (Pearson’s correlation coefficients from the current quantitative data), they were formed to serve as a latent construct of psychosocial stress (α = .71).

All of the above measures have been adapted from the tools by other authors as well as from our qualitative research.

## Methods

### Research site

The main site for this study was the city of Hanoi, located in Northern Vietnam. The current population is now 6.5 million. Hanoi is one of the cities with the highest HIV/AIDS prevalence in adults within Vietnam [1]. With its large area, industry and services, Hanoi is also one of the two largest cities in Vietnam and one of the most frequent choices for unkilled laborers, migrant laborers, and rural–urban migrants.

#### Sample size and participants

**Figure 2:**
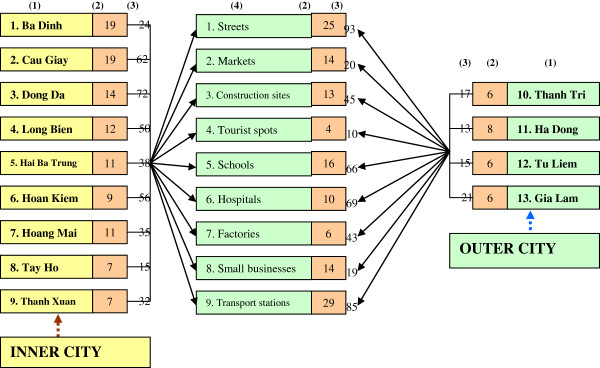
**Sample frame and size.** Notes: (**1**) District list, (**2**) Number of venues, (**3**) Number of respondents, (**4**) Type of venues 2.

Participants of the present study were male street laborers. Male street laborers were selected because they outnumbered female counterparts traveling to cities to search for substances [36]. They also serve as a bridging population linking core groups of higher HIV transmission risk (sex workers and injection drug users) and the general population (wives, lovers and sex partners. As we did not have a sampling frame, we applied a social mapping technique [37]. The purpose of this exercise was to identify as many venues as possible of male laborers - most being unskilled and unregistered working on the streets in districts of Hanoi. The districts were weighed by their level of social services concentration and urbanization. In this way, only urban and suburban districts where most of male street laborers congregated to search for casual jobs were mapped. In each district, trained field workers traveled to places where there was a high concentration of male street laborers. Typically this was in streets, markets, construction sites, transport stations (including railway, bus, and taxi stations), tourist spots, or by other social services - schools, hospitals, and factories. In each venue key informants such as street laborers, local people living close to the venue, local leaders, experienced researchers from prior studies on mobile populations, peer educators and outreach officers were consulted for mapping the next venue. During the mapping, field workers were also asked to estimate the number of potential participants. Afterwards, a list of all the venues (135 venues across 13 districts in Hanoi) and a total estimated number of participants were created. Between 3 to 6 venues in each district were randomly selected and all of the participants in each venue were approached for interviews. During the interviews, participants were screened if they were (1) male, (2) 18 to 59 years old, and (3) sought casual jobs or worked on the street, mostly low-skilled and unregistered, and (4) not interviewed before (to avoid duplication of interviews). The list of districts, types of venues and number of respondents included in the study are presented in Figure 2.

#### Survey procedures and ethics considerations

The research instrument was first validated on a sample of 55 participants. The pilot demonstrated that the instrument was technically suitable in terms of face validity and internal consistency (Cronbach’s alpha of most scales > 0.70) for the main survey. In total 450 eligible participants who provided informed consent completed interviews and were included in this study.

Participants were verbally informed about the study, that participation was voluntary, that they had the right to withdraw at any point, and, that data would be handled confidentially. After obtaining informed consent, an anonymous, structured questionnaire was administered to participants as a face-to-face interview. To limit external interference, interviews were conducted either in participants’ homes, in the home of researchers or a location convenient to participants. For those interviewed at worksites or on streets, permission was sought to interview participants separately. Well-trained interviewers and individualized interviews were also able to reduce the effect of the external environment. Each questionnaire took approximately 30–45 minutes to complete. Each participant was given AU$10 to compensate for his time. The study protocol was approved by the Institutional Review Boards at both Queensland University of Technology in Australia and Hanoi Medical University in Vietnam.

### Measures

The measures for the constructs of the original and modified model are presented in Table 1.

*Risky Drug Use Behavior* was assessed with five items [23] asking 1) if participants ever took a drug in their lifetime, 2) if they ever injected a drug in their lifetime, 3) how often did they inject drugs during the past month, 4) how often did they re-use syringes offered from other peers during the past month, and 5) how often did they offer their syringes to other peers during the past month. Items 3, 4 and 5 were then classified into a dichotomous scale with 0 being coded as no or a lower level of the respective practices, and 1 being labeled as a higher level of each practice. These items were summed to form a composite score of the level of risky drug use behavior (α = .82).

### Data analysis

SEM [38], the main procedure of statistical analysis, was conducted with data from 450 male laborers for a principal outcome variable of risky drug use. We adopted the Weighted Least Squares (WLS) estimation given that some of the variables in the model were not normally distributed. Model fit was assessed first with the p-value of WLS Chi square and then with the comparative fit index - CFI [39,40] and the root-mean-square error of approximation – RMSEA [41]. To be fit, WLS χ^2^ should be not significant (i.e. *P* > 0.05). The CFI ranges from 0 to 1, with .90 indicating acceptable fit and .80 indicating marginal fit [39]. The RMSEA ranges from 0 to ∞, with fit values less than .05 indicating close fit and less than .10 indicating fairly acceptable fit [40]. The CFI and RMSEA are sensitive to model misspecification and are minimally affected by sample size [42]. Both the original and modified IMB models were first tested separately, followed with an examination of their fit to which model is better to predict risky drug use.

## Results

### Sample characteristics and drug use patterns of male laborers

The mean age of male unskilled, unregistered laborers was 39 years. These men had a minimal education level (mean grade completed = 8; in Vietnam the education system classifies 12 grades ranging from 1 to 12 for primary, secondary and high school, and over 12 for higher education). Most were married (84%), migrant (87%), ethnic Kinh (~98%), Buddhist and ancestor worship followers (~66%), and rural workers (60%). The majority (~60%) were farmers in their hometown and the most common occupation during their urban stay was motorbike driver (~65%), followed by manual laborer and construction worker, each contributing more than 10% of the total. The average monthly income was 2.6 million VND (an equivalent of U.S.$130). The response rate was high, representing 95% of the participants.

As presented in Table 2, the prevalence of lifetime drug users was fairly high (over 17%), most (97.4%) of whom were injectors. Sharing injecting equipment among participants was quite common with 40% almost everytime and/or always re-using syringes and needles given by other users and 38.67% almost everytime and or always giving equipment to other users. 29.33% never and/or only once bought syringes and needles during the past month; 34.66% rarely and/or never kept syringes and needles available; and 35.33% rarely and/or never discussed or persuaded with peers not to share injecting equipment.

**Table 2:** Characteristics of drug use

**Variable (N = 450)**	$x\bar{}$ **± SD**	**N (%)**
Lifetime drug use (N = 450)		77(17.11)
Average age at first use (N = 77, range = 15-50)	26.95 ± 9.69	
Lifetime drug injection (77)		75(97.40)
Average age at first injection (N = 75, range = 16-51)	29.12 ± 9.79	
Injection use during the past month (n = 77)		
Frequency of injection (range = 0-6)*	3.4 ± 1.38	
None during the past month		2(2.60)
Less than monthly		6(7.80)
Around once per month		12(15.6)
A few times per month		26(33.76)
Weekly		17(22.08)
A couple of times per week		5(6.49)
Daily		9(11.69)
Sharing syringes and needles given by other users during the past month (N = 75)		
Frequency of sharing syringes and needs (range = 0-5)*	3.01 ± 1.31	
Never		1(1.33)
Rarely, seldom		15(20.00)
Sometimes		9(12.00)
About half of the time		20(26.67)
Almost everytime		23(30.67)
Always		7(9.33)
Giving syringes and needles to other users to share during the past month (N = 75)		
Frequency of sharing syringes and needs (range = 0-5)*	2.83 ± 1.31	
Never		1(1.33)
Rarely, seldom		16(21.33)
Sometimes		13(17.33)
About half of the time		16(21.33)
Almost everytime		24(32.00)
Always		5(6.67)
Purchasing syringes and needles during the past month (N = 75)		
Purchasing syringes and needles (range = 0-4)*	2.15 ± 1.06	
Never		3(4.00)
Once		19(25.33)
Sometimes		26(34.67)
Often		18(24.00)
Always		9(12.00)
Keeping syringes and needles available during the past month (N = 75)		
Frequency of keeping syringes and needles (range = 0-4)*	2.15 ± 1.17	
Never		4(5.33)
Rarely		22(29.33)
Sometimes		20(26.67)
Often		17(22.67)
Always		12(16.00)
Discussing or persuading peers not to share syringes and needles when injecting (N = 75)		
Frequency of discussing or persuading peers not to share (range = 0-4)*	1.75 ± 1.17	
Never		12(16.00)
Rarely		22(19.33)
Sometimes		19(25.33)
Often		17(22.67)
Always		5(6.67)

*Higher scores indicating higher levels of the practice or higher risk behavior.

### Descriptives of modified IMB model constructs

The means, standard deviations and intercorrelations between the scales and sub-scales included in the model are presented in Table 3. Mobility was low, alcohol consumption and depression levels were close to moderate, whilst social isolation levels were fairly low. Heuristic and transmission information levels were scored as medium, whilst attitudes, norms, and intentions were fairly positive ($x\bar{}$ = ~ 26; range = 7-35). Reported behavioral skills were also moderate to fairly high, whilst the magnitude of drug use was relatively high ($x\bar{}$ =2.81; range = 0-5). Regarding intercorrelations among sub-constructs, with the exception of some small correlations, the majority of the scales and subscales were moderately and closely related to one another (r’s = .30-.87; *P* < .05, <.01, and < .001); the correlations among sub-scales of psychosocial stress were moderate to robust (r’s = .14-.65; *P* < .05 and < .001). This suggests that scales and subscales demonstrated construct validity.

**Table 3:** Means and standard deviations and correlates among modified IMB model constructs

**Constructs**	$x\bar{}$ **± SD (Range)**	**1**	**2**	**3**	**4**	**5**	**6**	**7**	**8**	**9**	**10**	**11**	**12**
1.Mobility Index	.35 ± .77 (0–10)	-											
2.Alcohol Use	5.66 ± 4.83 (0–28.50)	.14	-										
3.Social Isolation	7.20 ± 3.79 (0–20)	.23*	.25*	-									
4.Depression	6.65 ± 5.16 (0–27)	.17*	.33*	.65***	-								
5.Heuristic Information	2.70 ± 1.04 (0–4)	- .17*	-.12	.14	-.10	-							
6.Transmission Information	1.5 ± 0.97 (0–3)	-.30*	.18	.14	-.02	.16	-						
7.Attitudes	26.40 ± 4.58 (10–35)	-.14	-.40**	.29*	-.20*	.60***	.31*	-					
8.Norms	26.35 ± 5.18 (8–35)	-.18*	-.33*	.29*	-.18*	.56***	.33*	.87***	-				
9.Intentions	25.85 ± 5.44 (7–35)	-.12	-.36**	.30*	-.32*	.59***	.38**	.83***	.87***	-			
10.Preparation	10.79 ± 2.63 (3–15)	-.21*	-.18	.30*	-.27*	.65***	.46**	.81***	.82***	.78***	-		
11.Practice	7.07 ± 1.90 (2–10)	-.27*	-.28*	.27*	-.18*	.60***	.41**	.81***	.84***	.81***	.87***	-	
12.Drug Use Level	2.81 ± 1.31 (0–5)	-.08	-.26*	.29*	-.19*	.54***	.44**	.73***	.75***	.72***	.75***	.77***	-

*P < .05; **P < .01; ***P < .001.

Skill 1 = Preparation; Skill 2 = Practice.

### Model estimation

**Figure 3:**
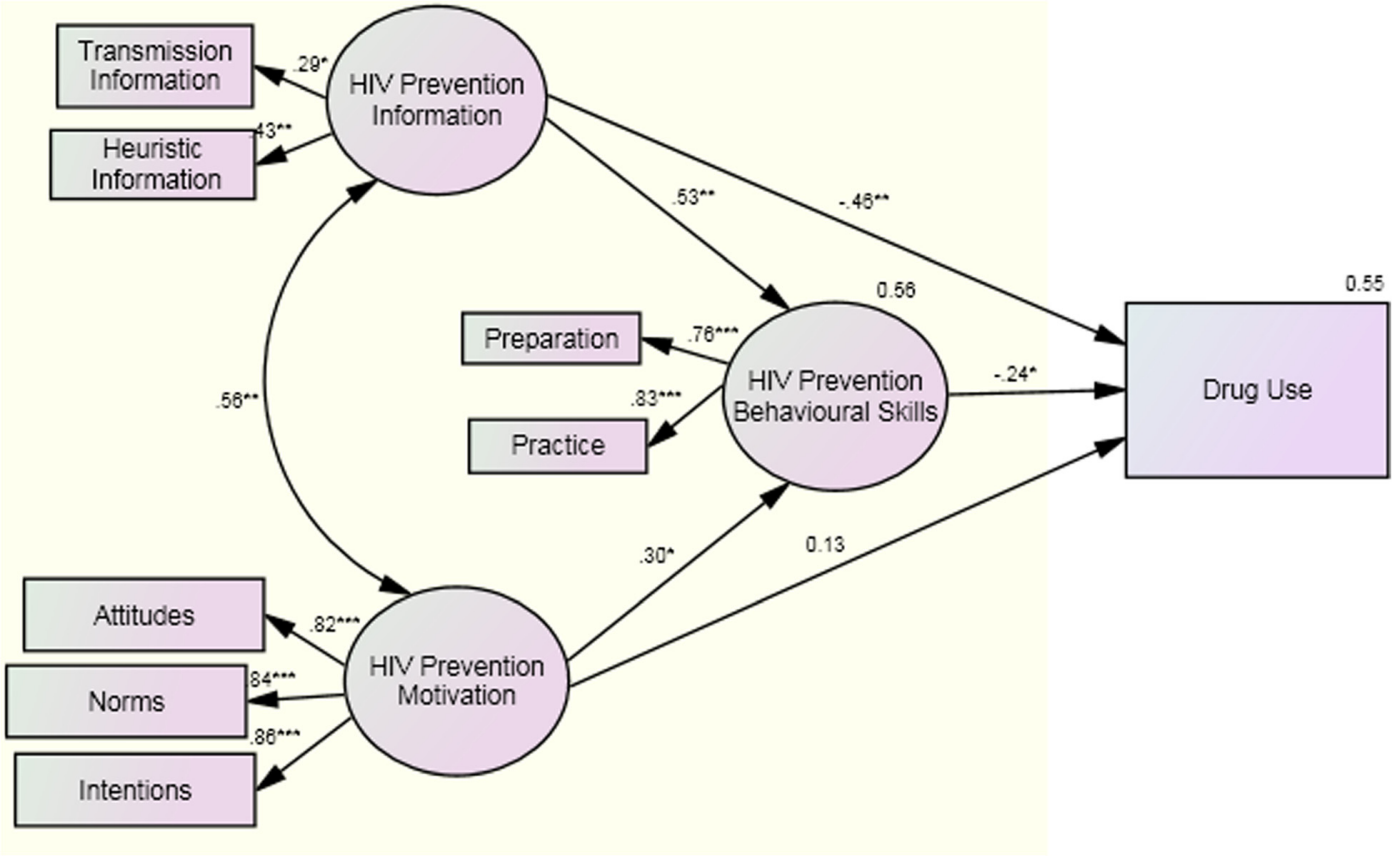
**Estimation of the IMB model of risky drug use behavior.** Notes: Coefficients are standardized path coefficients. Single-headed arrows represent one-way relationships, double-headed arrows covariates. Variables in eclipses represent latent variables, in squares observed variables. Overall model fit: ML χ2 (16, N = 450) = 15.52, P>.05; CFI = .95; RMSEA = .008. Paths: *P<.05; **P<.01; ***P<.001

Figure 3 displays constructs of the original IMB model estimated with standardized path coefficients for all estimated paths and loadings. A path coefficient is a standardized regression coefficient (beta) showing the direct effect of an independent variable on a dependent variable in the path model. The path coefficient of greater than .30 reflects at least a moderate relation between two variables. There was a significant path from information and motivation to behavioral skills (*β’s* = .53 and .30, respectively; *P* < .05), and from behavioral skills to drug use behavior (*β* = −.24; *P* < .05), indicating that individuals who were more informed and motivated to prevent HIV were more likely to have perceived behavioral skills necessary were less likely to engage in risky drug use behavior. There appeared to be no direct relationship between motivation and behavior, but there was a significant negative relationship between information and behavior. All associations among the sub-constructs were statistically significant. Fifty-seven percent of the variance in drug use was accounted for by the model. The indices of fit were satisfied (WLS χ^2^ = 15.52, *P >* .05; CFI = .95; RMSEA = .008).

**Figure 4:**
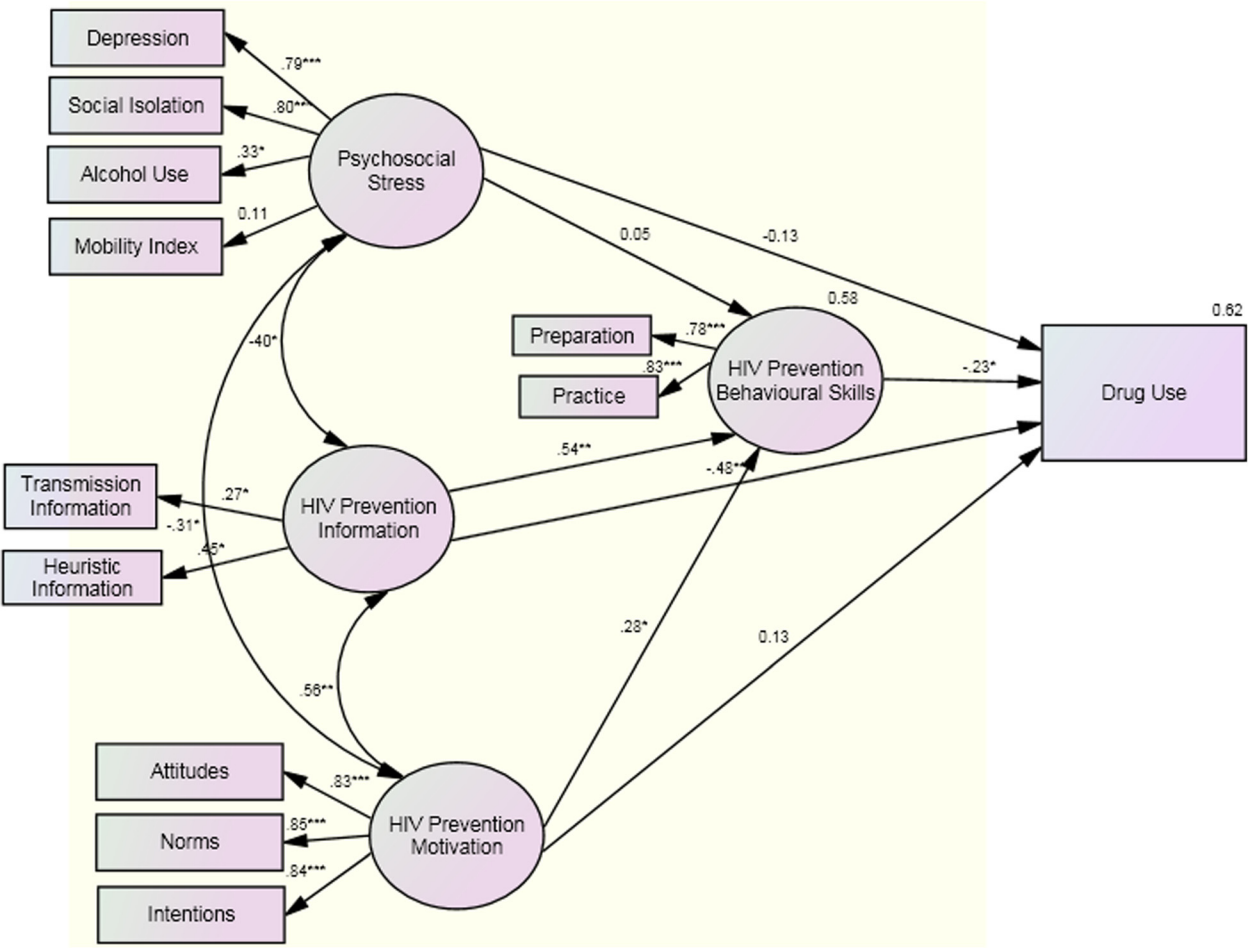
**Estimation of the modified IMB model of risky drug use behavior.** Notes: Coefficients are standardized path coefficients. Single-headed arrows represent one-way relationships, double-headed arrows covariates. Variables in eclipses represent latent variables, in squares observed variables. Overall model fit: ML χ2 (46, N = 450) = 101.12, P<.05; CFI = .91; RMSEA = .06. Paths: *P<.05; **P<.01; ***P<0.001.

Figure 4 shows standardized path coefficients for all estimated paths and loadings of the constructs of the modified IMB model. All of the paths from psychosocial stress and motivation to behavior were statistically not significant (β’s = −.13 & .13, respectively, *P* > .05). The path coefficient from psychosocial stress to behavioral skills was also not significant (β = .05, *P* > .05). However, there were statistically significant paths from information and motivation to behavioral skills (β’s = .54 & .28, respectively, *P <* .05), and the path coefficient from information to behavior was also significant. The relationship between behavioral skills and behavior was significant (β = −.23; *P* < .05). All but one path from the main construct to sub-constructs – psychosocial stress to mobility index - were statistically significant. Besides the psychosocial stress, other factors such as education level, urban/rural origin, type of work during urban residence, marital status, ethnicity, religion, and with whom participants live during urban stay, were examined, but no significant change in the model was identified (data not shown). In this model, sixty percent of the variance in drug use behavior was accounted for by the constructs. Nevertheless, the model was not fit [WLS χ^2^ (46, N = 450) = 101.12, *P =* <.05; CFI = .91; RMSEA = .06].

The mediation effect of behavioral skills in the IMB model continued to be examined (data not shown in the interest of space). When we removed two paths from information and motivation to behavior, the path coefficient from information to behavioral skills was still significant (β = .47, *P* < .05), the path coefficient from motivation to behavioral skills was increased (β = .33, *P* < .05), and the path coefficient from behavioral skills to behavior significantly increased (|β| = .49, *P* < .01). When we removed two paths from information and motivation to behavioral skills, path parameters from information and motivation to behavior appeared unchanged. These data suggest that behavioral skills was a complete mediator between information, motivation and behavior.

## Discussion

In this study among male street laborers, most of whom were rural-to-urban migrant, low-skilled and unregistered, over 17% were drug users. Compared with other populations, the proportion of drug users in our sample was much higher. More than 10% of Vietnamese youths aged 15–24 in Quangninh province used drugs [43], almost 11% of the general population in urban Thailand similarly use [44], and the rate among several communities in some areas of rural and urban China was 1% [5]. Unfortunately, data on the drug use behavior identified in this present study are not comparable in Vietnam given the lack of previous research examining this issue among rural–urban migrant low-skilled workers. With regards to injecting risk behaviors, our data is quite consistent with studies by Lurie et al. [45] of drug users in some parts of Africa, by Deren et al. of Puerto Rican drug users in the New York [46], and Yang, et al. of drug users in southwestern China [47] demonstrating that needle sharing was not uncommon. As reported by Deren et al. [46], over one-third of American injectors shared syringes or other paraphernalia associated with HIV and hepatitis C (HCV) transmission (cookers, cotton, water), and 15% used shooting galleries. Similarly, according to data by Yang et al. [47], close to 60% of the sample of drug users injected drugs, and 35% of those who injected drugs shared used needles when injecting during the past 30 days in China.

The current study also found that male street laborers showed moderate knowledge and understanding of HIV/AIDS and the risk behaviors associated with transmission. They were also moderately motivated and reported fairly high behavioral skills to prevent HIV transmission, but still engaged in risk behaviors related to injecting drugs. The findings of this study seem to support previous data. For instance, heroin users in American methadone maintenance programs [26], adolescent substance users in the US [48], and truck drivers in India [49] had a relatively moderate understanding of HIV theory, displayed a medium level of motivation, including attitudes, norms and intentions, and reported perceived higher behavioral skills for HIV prevention, but practiced a drug use risk behavior at high level.

The implications and application of the findings from this study can be understood within the context of the theory of HIV prevention-related IMB model which largely reflects psychological determinants of HIV/AIDS prevention behaviors [10,11]. According to this theory, HIV/AIDS prevention behaviors are a function of information, motivation, and perceived ability of behavioral skills concerning those behaviors. However, studies on different populations (excluding male street laborers) using this theoretical framework have produced mixed results [16]. Fisher et al. and Carey et al. [50,51] held that studies for confirmation dealing with very diverse populations remain limited, whereas Odutolu [16] claimed that the model focused heavily on individual and psychological factors, neglecting other social contexts. In our current study, we examined a modified version of the IMB model. Overall, the modified model is likely to have a robust prediction of drug use behavior at high risk for HIV, as sixty percent of the variance in the behavior was accounted for by the model. However, this modified model (total variance = 62%, *P* (WLS χ^2^) < .05; CFI = .91 & RMSEA = .06) was not of adequate fit compared with the original version (total variance = 55%, *P* (WLS χ^2^) > .05; CFI = .95 & RMSEA = .008) in predicting the behavior. This IMB model contributed up to 55% of the variance in the behavior, approaching the upper limit of percentage variance in the outcome variables as compared to other behaviors and populations [11,13,21,26,52-55] (see Table 4). This model revealed that the effects of information and motivation on drug use behavior were completely mediated by behavioral skills. There was a significant effect of information (β = .53, *P* < .01) and motivation (β = .30, *P* < .05) on behavioral skills which, in turn, significantly predicted a lesser likelihood of risky drug use behavior (β = −.23, *P* < .01). Examination of the significance of the mediated effect showed that there was a significant total indirect effect of information and motivation on drug use behavior through a combination of IMB constructs (*P* of z-test < .05). This suggests that male street laborers who are more informed and motivated are more likely to report better behavioral skills, which in turn, are less likely to be engaged in a risky drug use behavior. Any change in behavioral skills appears predict risk behavior such as drug use. Our findings appeared to support the original version of the IMB model as a better predictor of HIV-related risk or protective drug use behavior. One possible explanation for this would be that other broader environmental and social factors such as structural social capital and social policies may also be influences on drug use. According to Harpharm et al. [56], fewer drug users have been related to having actual participation in communities, institutional linkages with services, facilities and organizations, frequency of general collective action, specific collective action and other connections. Recognizing the important role of material social capital in shaping risky drug use behavior allows researchers and policy makers to think about how to better inform policies for preventing risky drug use behavior among male street laborers.

**Table 4:** Comparison of percentage variance across various populations

**Sample**	**Outcome variable (model version)**	**References**	**Percentage variance in outcome variable**
Male street laborers	Drug use behavior (Modified IMB)	Our current study	57
Male street laborers	Sexual behavior (Modified IMB)	[54,55]	58
Indian truck drivers	Sexual behavior (IMB)	[21]	40-51
Heroin addicts	Sexual behavior (IMB)	[26]	35
Urban minority high school males	Sexual behavior (IMB)	[12]	75
Urban minority high school females	Sexual behavior (IMB)	[12]	46
Low-income African American females	Sexual behavior (IMB)	[13]	36
Low-income white females	Sexual behavior (IMB)	[13]	57
Netherlands adult homosexual males	Sexual behavior (IMB)	[53]	26
Heterosexual university males and females	Sexual behavior (IMB)	[11]	10
Homosexual adult males	Sexual behavior (IMB)	[11]	35

Given the current results, it is recommended that a sound HIV control program targeting this population not only distribute information and enhance motivators (attitudes, norms, and intentions) for HIV prevention, but also consider interventions that could improve self-efficacy or behavioral skills in order to increase drug use-related preventive behaviors or reduce risky behaviors for HIV. The findings highlight an important point for designing intervention programs for these men. For a high risk behavior as injection drug use, it appears to be essential to focus upon individual and psychological factors, while it may also be helpful to investigate broader environmental and social factors that would contribute to drug use.

This study has some limitations. Its cross-sectional design may have precluded the ordering of causality. Self-report bias was also possible due to the social unacceptability of drug use. Since there were some questions that required respondents’ recall, recall bias may be unavoidable. The construct validity of the variables in the model has been examined based on Pearson’s product moment correlation statistics between pairs of variables and the results of factor analysis for the scales used in the model. As Vietnam has many cities that resemble Hanoi, the results of this study could be helpful to other similar urban settings.

However, these limitations notwithstanding, the study provides some significant insights. As it is the first study to examine the fit of the IMB model with this under-researched population, it contributes to our understanding and literature. Further, most of the model constructs were measured with multiple items which were assessed with adequate reliability. Finally, as this is a preliminary investigation, this leads us to a number of interesting implications for further research and intervention in this area. Interventions designed for this population should seek to address informational and motivational impediments to a change in risky drug use behavior as well as improve behavioral skills which help reduce risky drug use behavior. Future research that uses intervention designs with longitudinal follow-up will be crucial for determining causal ordering of the model constructs. There is also a need for further examination of the modified model of IMB in relation to the original version in other populations in order to support interpretations of model fit and consistency. The modified model may include broader environmental and social factors which contribute to risky drug use.

## Conclusions

Overall, this research is a first step toward further research into high risky drug use behavior and factors that may fuel the HIV epidemic among such men. The research is helpful in building an increased understanding of the risks for HIV infection and transmission among male street laborers enabling policy makers and practitioners to deal with this uncertain, disturbing, and increasing epidemic. An AIDS prevention program for these men should not only distribute information and enhance motivations for HIV prevention, but consider interventions that could improve self-efficacy for preventing HIV infection. Future public health research and action may also consider broader factors such as structural social capital and social policy to alter the conditions that drive risky drug use among these men. As Hanoi has much in common with many other rapidly urbanized cities in Vietnam, this research provides evidence, policy and practical implications that can be useful to urban settings within the country.

## Abbreviations

IMB: Information-Motivation-Behavioral Skills model; SEM: Structural equation modeling; VND: Vietnam Dong (the Vietnamese currency); WLS: Weighted Least Squares.

## Competing interests

We declare that we have no competing interests.

## Authors’ contributions

NVH designed the study, wrote the protocol, conducted fieldwork, analyzed data, wrote and revised the manuscript. MPD reviewed the protocol, adviced on the manuscript and edited the language. JD reviewed the protocol, reviewed the manuscript and edited the language. All authors read and approved the final manuscript.

## Authors’ information

*HVN*, a Master of Health and International Development, a PhD in Public Health, is a lecturer and a researcher of the Department of Health Management and Organization, Institute for Preventive Medicine and Public Health, Hanoi Medical University (HMU), Vietnam.

*DPM*, a PhD, a Professor of Social Epidemiology, is a senior lecturer of the School of Public Health and Social Work at Queensland University of Technology in Brisbane, Queensland, Australia, and a director of Vietnam-Queensland University of Technology Public Health Cooperation Program, Australia.

*DJ*, a PhD, an Associate Professor, is a researcher at Brisbane Sexual Health and HIV Service, MetroNorth Hospital and Health Service, in Brisbane, Queensland, Australia.

## Pre-publication history

The pre-publication history for this paper can be accessed here:

http://www.biomedcentral.com/1471-2458/13/453/prepub
